# Major enteropathogens in humans, domestic animals, and environmental soil samples from the same locality: prevalence and transmission considerations in coastal Odisha, India

**DOI:** 10.4178/epih.e2020034

**Published:** 2020-05-26

**Authors:** Arpit Kumar Shrivastava, Nirmal Kumar Mohakud, Swagatika Panda, Saumya Darshana Patra, Subrat Kumar, Priyadarshi Soumyaranjan Sahu

**Affiliations:** 1 Infection Biology Laboratory, School of Biotechnology, Kalinga Institute of Industrial Technology (KIIT) Deemed to be University, Bhubaneswar, India; 2 Kalinga Institute of Medical Sciences, Kalinga Institute of Industrial Technology (KIIT) University, Bhubaneswar, India; 3 Department of Microbiology and Immunology, Medical University of the Americas, Nevis, West Indies

**Keywords:** Diarrhoea, Molecular epidemiology, Coinfection, Zoonoses, India

## Abstract

**OBJECTIVES:**

Regions with limited sanitation facilities have higher rates of infections with various enteric pathogens. It is therefore important to identify different hosts and their relative contribution to pathogen shedding into the environment, and to assess the subsequent health risks to humans.

**METHODS:**

In this study, human faecal (n=310), animal faecal (n=150), and environmental (soil) samples (n=40) were collected from the same locality and screened for selected enteric pathogens by immunochromatography and/or polymerase chain reaction.

**RESULTS:**

At least 1 microbial agent was detected in 49.0%, 44.7%, and 40.0% of the samples from human, animals, and soil, respectively. Among humans, rotavirus was predominantly detected (17.4%) followed by enteropathogenic *Escherichia coli* (EPEC) (15.4%), *Shigella* (13.8), and Shiga toxin-producing *E. coli* (STEC) (9.7%). Among animals, STEC was detected most frequently (28.0%), and EPEC was the major enteric pathogen detected in soil (30.0%). The detection rate of rotavirus was higher among younger children (≤2 years) than among older children. Single infections were more commonly detected than multiple infections in humans (p<0.01), unlike the observations in animal and soil samples. For diarrhoeagenic *E. coli* and *Shigella*, most of the human and animal isolates showed close relatedness, suggesting possible cross-infection between humans and domesticated animals in the area studied.

**CONCLUSIONS:**

The present study provides an improved understanding of the distribution of major enteric pathogens coexisting in humans and animals in the region, thereby suggesting a high potential for possible transmission among livestock and communities residing in the studied locality.

## INTRODUCTION

The burden of gastrointestinal infections remains a major problem, especially in low-income countries, as worldwide data show that infectious diarrhoeal disorders alone account for nearly 0.8 million deaths in children less than 5 years of age annually [1]. Multiple aetiological agents, including bacteria, viruses, and parasites, contribute to the diarrhoea of infective aetiology in humans and animals [2]. These diarrhoeal agents are most commonly transmitted either through contaminated food and water or through the faecal-oral route. Therefore, lack of access to clean water, insufficient hygiene, and inadequate sanitation conditions in resource-poor settings put the community at high-risk of suffering from diarrhoea [3]. The anthroponotic and zoonotic transmission of diarrhoeal diseases occurs through a wide range of environmental reservoirs contaminated with various diarrhoeagenic pathogens common to both humans and animals [4]. In particular, domestication of livestock and pet practices often contribute to the zoonotic transmission of intestinal pathogens through faecal contamination of bodies of water [5]. The soil might also play a major role in transmission of enteric diseases, but this crucial link to infections has not yet been well studied [6].

Frequent foodborne and waterborne outbreaks of infectious diarrhoea have encouraged microbiologists and epidemiologists to conduct ecological studies to understand the zoonotic and anthroponotic transmission of various diarrhoeal pathogens. In community settings in India, diarrhoeal syndromes are characterized by high faecal shedding, infectivity, growth, persistence, exposure to site-specific environmental conditions, pathogen detection, and faecal contamination [7]. However, only limited information exists regarding the association between domestic exposure and zoonotic transmission of genetically diverse diarrhoeal pathogens in the eastern part of India. A pilot survey was therefore conducted in an eastern coastal province in India in order to investigate the prevalence and possible genetic diversity of major diarrhoeal agents in faecal samples from symptomatic humans, domestic animals, and soil samples from the same locality through microbiological investigations.

## MATERIALS AND METHODS

### Study design and sample collection

**Figure 1.**
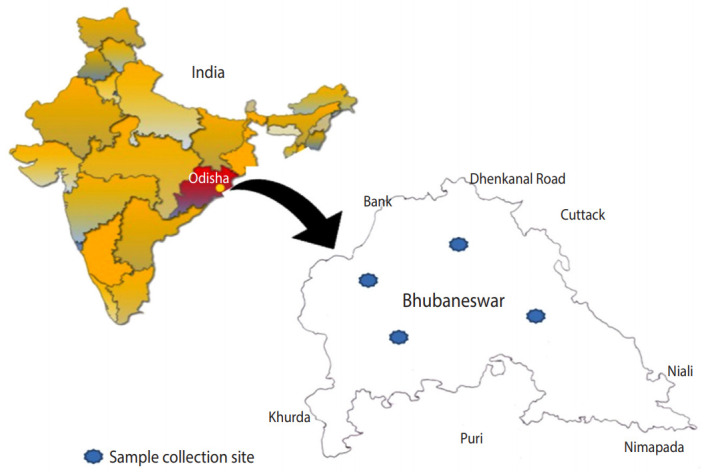
Sample collection site. The geographic location of Odisha is marked in red on the map of India. Samples were collected from four different zones blue colored regions in Bhubaneswar map showed sample collection site.

The present cross-sectional observational study was conducted in and around the city of Bhubaneswar, located at 20.27°N 85.84°E (Figure 1) in the state of Odisha, India. Human/animal faecal samples and soil samples were collected over 13 months (March 2016 to April 2017) [8]. Samples were collected from the Khurdha district, and humans and animals of all ages were considered for this study. In Odisha, open defecation is still practiced in rural areas and even in poor and urban slum communities in urban areas such as Bhubaneswar. The most preferred site for open defecation is near ponds or paddy fields. However, people in the community and animals frequently use pond water for bathing, drinking, and other recreational activities, and farmers similarly often visit paddy fields for irrigation and other farming practices. Consequently, these sites are the highest-potential areas for anthroponotic or zoonotic transmission of enteric pathogens. Therefore, we collected samples from ponds and paddy fields.

#### Human sampling

In total, 310 diarrhoeal human faecal samples were collected from 3 local hospitals and 2 local community clinics located a wide distance apart from each other across the study territory.

#### Animal sampling

Fresh faecal samples (n = 150) from symptomatic domestic animals (cattle, sheep, and goats) were collected across the study region. To minimize the risk of environmental contamination, fresh faeces was carefully collected from the surface of the mass that had no direct contact with the soil.

#### Soil sampling

Forty soil samples from paddy fields and banks of ponds were obtained. After collection, the samples were placed in appropriate boxes with ice packs and transported to the laboratory within 4 hours of collection. The samples were stored temporarily in a refrigerator at 4°C, and each sample was processed within 24 hours of collection.

### Immunochromatographic test

Faecal specimens collected from symptomatic human and animal subjects were screened for rotavirus and adenovirus by an immunochromatographic test (Combi-Strip C-1004; Coris Bioconcept, Gembioux, Belgium) following the manufacturer’s instructions.

### Genomic DNA extraction and quantification

Total faecal genomic DNA from human and animal faeces was extracted from the stool using the QIAamp Fast DNA Stool Mini Kit (Qiagen, Hilden, Germany) and soil DNA was extracted using MP Biomedicals FastDNA SPIN Kit for Soil (MP Biomedicals, Burlingame, CA, USA) following the manufacturer’s instructions.

### Polymerase chain reaction amplification and sequence analysis

Polymerase chain reaction (PCR)-based detection was employed for various possible microbial agents. Separate primer sets were used for target-specific amplification of each microbial agent, as presented in Supplementary Material 1. For each pathogen, genomic DNA extracted from stool (Qiagen Stool DNA kit) was used as a template for PCR amplification. Genomic DNA extracted from pure culture of each microbial agent was used as a positive control in PCR screening. The PCR cycling conditions for the targeted bacterial, viral, and protozoan diarrhoeal agents were as follows: initial denaturation at 95°C for 5 minutes, followed by 34 cycles of denaturation of 94°C for 30 seconds, annealing at a primer-specific temperature at 30-45 seconds, extension at 72°C for 1 minute, and final extension for 72°C for 7 minutes. All the PCR assays were equally sensitive and specific across all different sample types, and we used the previously validated primer sets presented in Supplementary Material 1. All PCR products were subjected to 1.0-1.5% agarose gel electrophoresis to confirm the positive samples. All PCR-positive products were purified and sequenced.

### Phylogenetic analysis

The sequences obtained from this study ([MF329642], [MF329643], [MF329644], [MF329645] [MF329646], [MF329647], [MF329648], [MF329649], [MF329650], [MF329651], [MF329652], [MF329653], [MF329654], [MF329655], [MF329656], [MF329657], [MF329658], [MF329659], [MF329660], [MF329661], [MF329662], [MF329663], [MF329664], [MF329665], [MF329666], [MF329667], [MF329668], [MF329669], [MF443209], [MF443210], [MF443211], [MF443212], [MF443213], [MF443214], [MF443215], and [MF443216]) and a few reference sequences from GeneBank ([HQ324789.1], [JQ407725.1], [AB630325.1], [JQ407711.1], [EU867486.1], [HM588724], [FR849543], [KT326927], [KY243935], [KX909565], [KU201272], [LT717486], [KP116114], [Z47381], [KP116115], [KP116113], [KP116116], [EU032322], [KF679722], [AY204229], [AY204227], [L16997], [AF159110], [JN812214], [KM199753], [AB441688], and [KM199745]) were compared for genetic relatedness. A neighbour-joining algorithm was implemented to construct a phylogenetic tree using Molecular Evolutionary Genetics Analysis version 6.0 [9].

### Statistical analysis

On the basis of descriptive statistics, odds ratios (ORs), 95% confidence intervals (CIs), and p-values were calculated to estimate significance. The chi-square statistic was calculated using a 2 × 2 contingency table in MedCalc (MedCalc, Osted, Belgium). Principal component analysis was done using METAGENassiat [10] to analyse the possible clustering patterns of human infections acquired from animal and environmental sources.

### Ethics statement

The study protocol was reviewed and approved by the Institutional Ethical Committee of the Kalinga Institute of Medical Sciences. Informed consent and patient datasheets were maintained for all human participant.

## RESULTS

A total of 152 of 310 (49.0%) human samples, 67 of 150 (44.7%) animal samples, and 16 of 40 (40.0%) soil samples were found to be positive for at least 1 diarrhoeal pathogen. In the animals, the overall diarrhoeal pathogen detection rate was highest in sheep (41.1%), followed by goats (35.5%) and cattle (33.3%). Diarrhoeagenic *Escherichia coli* (DEC) was the major enteric pathogen detected in humans (28.7%), animals (38.7%), and soil (32.5%) (Table 1).

In humans, rotavirus was detected in 17.4% of cases, followed by enteropathogenic *E. coli* (EPEC) (15.5%) *Shigella* (13.9%), Shiga toxin-producing *E. coli* (STEC) (9.7%), enterohemorrhagic *E. coli* (EHEC) and enteroaggregative *E. coli* (EAEC) (4.5%), *Cryptosporidium* and adenovirus (3.9%), and *Giardia* (0.6%) (Table 1). In animals, STEC (28.0%), EPEC (14.7%), and EHEC (14.0%) were the major types of DEC detected, followed by *Cryptosporidium* (10.0%), adenovirus (4.7%), *Shigella* (3.3%), and *Giardia* (0.7%) (Table 1). In the samples, EPEC (30.0%) was the major enteric pathogen detected in soil samples, followed by *Shigella* (25.0%), STEC (15.0%), *Giardia* (7.5%) and *Cryptosporidium* (5.0%) (Table 1).

Sheep were found to be slightly more infected with DEC (41.7%) than goats (35.5%) and cattle (33.3%) (Table 2). *Cryptosporidium* were more often observed in goats (17.8%), while *Shigella* infection was predominant in sheep (6.7%). However, cattle were more likely to be positive for adenovirus than sheep and goats (Table 2). The distributions of diarrhoeal pathogens by age in humans and animals are shown in Table 3.

In our study, we observed coinfections with different combinations of bacterial, viral, and protozoan pathogens in both faecal and soil samples. Pathogens were detected simultaneously in 39.5% of human samples, 61.2% of animal samples, and 81.2% of soil samples (Table 4). Multiple pathogens were detected significantly more frequently in soil samples (p=0.009), followed by human samples (p=0.003) and animal samples (p=0.030). In humans, *Shigella* and STEC (8.5%) was the most common coinfection followed by rotavirus and EPEC (7.2%) and *Shigella* and EPEC (6.6%) (Table 4). The most frequent combination in animals was EHEC and STEC (23.4%) (Table 4). From all positive animal samples, we observed the highest percentage of coinfections in sheep (70.8%), followed by cattle (50.0%) and goats (37.5%). The combinations of *Shigella* and EPEC (37.5%) and STEC and EPEC (31.2%) were predominant in soil samples (Table 4).

Phylogenetic analysis was done to investigate the genetic relatedness and evolutionary dynamics of the strains circulating between humans and animals in the study region. Phylogenetic trees were constructed separately for each group of pathogens (Supplementary Materials 2-5). Among the DEC strains, STEC, EPEC, EHEC, and EAEC clustered in individual nodes isolated from humans and animals were found close to each other (Supplementary Material 2). Similar patterns were observed in *Shigella* isolates from humans and animals (Supplementary Material 3). *Cryptosporidium* and adenovirus isolates showed close relatedness with other strains that were isolated from domestic animals, birds, or environmental samples (Supplementary Materials 4 and 5).

**Figure 2.**
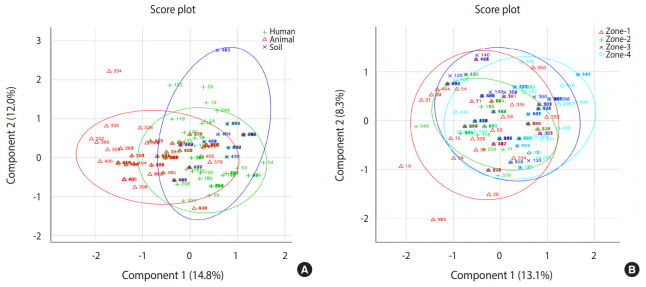
Principal component analysis. A total of 500 samples were analyzed to see the pattern of diarrheal infectious agents in study region, samples were calculated at 95% similarity. Two samples position in score plot close to each other are more alike and samples positions are far away are dislike from each other. (A) Score plot represents the presence of diarrheal agents in humans, animals and soil samples. Overlapping area in plot represents the similar pattern of infectious agents. (B) Zone wise pattern of infectious agent presented in the study area. Most of the samples share common overlapping zones, which showed all detected pathogens are present almost equally in each study zone.

Based on principal component analysis, 3 different clusters were generated for the human, animal, and soil samples representing the patterns of infectious agents at the genus level in the study region (Figure 2). All 3 groups shared a large portion of genera, revealing that the distribution of infections in human, animal, and soil samples was comparable. Samples collected from 4 different zones showed very similar patterns of distribution of infectious agents. All detected pathogens were distributed throughout the study area.

## DISCUSSION

Many resource-poor or developing countries have limited sanitary infrastructure, accompanied by a lack of awareness among communities that are suspected to be deprived of adequate education and awareness. Irrespective of focal urbanization and development of sanitation facilities, communities in underdeveloped pockets in and around urban areas are commonly reported to have higher rates of infections, particularly those associated with enteric pathogens [11]. This also places urban populations at a higher risk of acquiring infections because of their dependence on the communities living in the outskirts. In the present study, at least 1 diarrhoeal agent was detected in 40–50% of environmental samples and samples from animals and humans. When comparing the isolates from these 3 sources, we observed genetic similarities among the isolates, indicating the possibility of circulation of these microbial agents among humans, animals, and the environment in the study region.

Pathogenic *E. coli* was present in 32.5% of the soil samples. The detection of pathogenic *E. coli* in soil was also reported in another recent study from Kenya [12]. Hence, it is important to evaluate the environmental sources (soil and water) that might play an important role in retaining diarrhoeal pathogenic agents and act as a source of infection transmission, affecting both humans and animals.

Cattle and other ruminant animals might serve as reservoirs of STEC strains that are potentially pathogenic in humans [13,14]. In our study, STEC was detected most frequently in cattle (26.7%), followed by soil (15.0%) and human samples (9.7%). Similar patterns were observed in a previous study from Tanzania, although the prevalence rates were lower (cattle, 9.0%; humans, 3.2%; and soil, 0.8%) [15].

Open defecation by animals and humans is a major contributor to microbial shedding into the environment (soil and water). Thus, various microbial agents from the soil can contaminate nearby community bodies of water, thereby exposing both humans and animals. Therefore, the soil can be a potential mode of transmission of diarrhoeal pathogens in low-income countries; Pickering et al. [16] were able to isolate pathogenic *E. coli*, enterovirus, rotaviruses, and human Bacteriodales from soil samples. In our study, we detected *Shigella*, EPEC, STEC, *Cryptosporidium*, and *Giardia* isolates in soil samples from the locality, while animal and human diarrhoeal cases were also found to harbour similar aetiological agents. Sequencing of the human EPEC and EHEC virulence genes *eaeA* and *aggR* showed similarities with the corresponding animal isolates. In contrast, the *Cryptosporidium* 18s rRNA sequence and adenovirus hexon gene sequence were similar to other *Cryptosporidium parvum* and adenovirus strains, respectively, that were isolated from domestic animals, birds, or environmental samples [17,18].

Molecular epidemiological studies of pathogenic *E. coli* have suggested that cattle, sheep, and goats are potential sources of diarrhoeagenic EPEC, EHEC, and STEC [5,19-21]. In our study, the frequency of detection of STEC, EPEC, and EHEC was higher in sheep than in goats and cattle. Similar results were reported in another study from Turkey, where the isolation rate of STEC, EPEC, and EHEC was higher in sheep and goats [22]. STEC have been found to be closely related genetically when isolates from cattle [23] and sheep [24] were compared. Our study showed the presence of STEC in both humans and animals, suggesting possible zoonotic transmission of this pathogenic strain of *E. coli*.

According to the results of the present study, *Shigella* was the third most common aetiological agent detected in symptomatic humans and animals in the study region. *Cryptosporidium* and *Giardia* were the other major enteric pathogens detected in all 3 sources, while adenovirus was detected in humans and animals only. Odagiri et al. [25] reported that adenovirus, *Giardia*, and *Cryptosporidium* were found in rural India with a higher prevalence than in our study.

In comparison to a single pathogen, the presence of multiple diarrhoeal pathogens might cause more severe diarrhoea and disease pathogenesis [26]. In one of our previous studies, we detected multiple diarrhoeal pathogens, similar to the findings of other studies [27]. In the present study, multiple diarrhoeal pathogens were detected significantly more often in humans, animals, and soil samples than single pathogens. The most common pairs of concurrent pathogens in this study were *Shigella* and STEC in humans, EHEC and STEC in animals, and STEC and EPEC in soil samples. Given the paucity of data on the rates of specific coinfections with multiple diarrhoeal pathogens in human, animal, and soil samples, it is difficult to say whether our data are within the expected range. This is certainly an area that needs further investigation to obtain a better understanding of patterns of coinfection and their associations with disease transmission dynamics.

In order to understand the genetic relatedness between the isolated pathogenic strains, sequencing was carried out and a phylogenetic tree was constructed. The phylogenetic tree showed similarities between the human, animal, and soil isolates. We observed that few DEC strains, such as EPEC, EAEC and EHEC, and *Shigella* shared similar genetic sequences and clustered under the same branch. This suggests the possible zoonotic transmission of DEC and *Shigella* between humans and domesticated animals in the study area. A phylogenetic tree analysis of *Cryptosporidium* and adenovirus found that these isolates showed sequence similarities with previously isolated human and animal strains.

Livestock is an important reservoir for a number of enteric pathogens that can affect human and animal health. A recent study suggested that 15 major enteric pathogens are responsible for zoonotic transmission in low-income and middle-income countries (LMICs), of which 5 enteric pathogens cause approximately 1 million annual deaths [28]. Systematic reviews have demonstrated that after the introduction of improved sanitation in LMICs, a 30-40% decrease in childhood diarrhoea occurred [29,30]. Interventional sanitation efforts may reduce the quantity of human excreta in the environment, but animals are still often present in the domestic environment in LMICs, and people in these countries may have frequent contact with them [31-33]. Thus, contamination from animal faeces may still contribute to a substantial burden of disease in humans. This study demonstrated the coexistence of potential diarrhoeal enteric pathogens in human, animal, and soil samples in the study region, suggesting the possibility of zoonotic and anthroponotic transmission.

Successive federal government programmes have emphasised building toilets to end open defecation. The current programme, the Swachh Bharat Mission, aims to provide sanitation to all households to end open defecation by October 2019. Prior to the launch of the cleanliness campaign, the coverage of sanitation in the state of Odisha was a mere 10.9%, and after all the efforts of the last 3 years sanitation coverage reached upto 70%, Odisha remains amongst the lowest-performing states, with nearly one-third of the population still not having access to toilets [34,35]. The present study area included many underdeveloped pockets in and around the city of Bhubaneswar, where access to improved sanitation is poor. This results in the practice of open defecation by large segments of the population. Direct dispersal of animal excreta into the environment is also common throughout the study region. This increases the potential risk of transmission of faecal pathogens in exposed communities. The present findings provide preliminary evidence of the diversity of potential possible transmission patterns of bacterial, viral, and protozoal diarrhoeal pathogens and provide an improved understanding of the distribution of these pathogens in humans, animals, and the shared environment (soil). Overall, this study will be helpful for expanding our knowledge of disease transmission in this region, so that transmissible diseases of concern can be controlled, thereby enhancing quality of life for the community.

Although the present observational study deployed a unique approach to study both animal and human pathogens from the same locality, there are still some limitations, particularly in the choice of environmental samples and the sample numbers. As discussed previously, only soil samples near ponds or paddy fields were investigated based on the assumption that these are the most preferred sites for open defecation, as locations where both human and animals visit frequently for various daily activities. The initial plan to include similar numbers of human/animal faecal and soil samples could not be fulfilled due to various reasons, including but not limited to funding, time constraints, and the exclusion of a few samples due to unavoidable technical errors during transportation from the field to the analysing laboratory. To better explore the role of zoonotic transmission, pairing of human faecal, animal faecal, and soil samples collected from the same locality would be valuable, as would information on animal ownership and contact with livestock; however, these factors went beyond the scope of this study. Therefore, larger and longitudinal cohort studies of infants, children, adults, animals, soil, and community water sources would provide improved estimates of the prevalence of these diarrhoeal pathogens and their transmission in the community.

**Figure 3.**
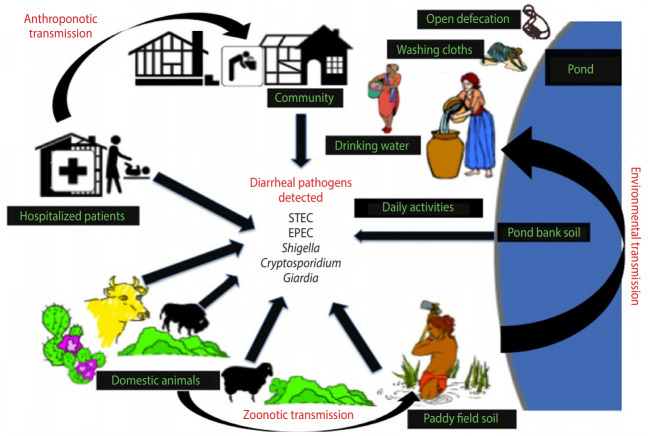
Possible routes of major diarrheal pathogens in the study area. STEC, Shiga toxin-producing *Escherichia coli*; EPEC, enteropathogenic *E. coli*.

The present study provides an improved understanding of the distribution of major enteric pathogens coexisting in humans and animals in the region, thereby suggesting a high potential for transmission among livestock and communities residing in the studied locality via contaminated soil and/or water (Figure 3). Future research on zoonotic and anthroponotic transmission of faecal contaminants should involve host-specific markers to determine the precise pathways of pathogen transmission in the region.

## Figures and Tables

**Table 1.** Frequency of detection of diarrhoeal pathogens in humans, animals, and soil samples

Infectious agent	Humans (n=310)			Animals (n=150)			Soil (n=40)		
	Positive, n (%)	OR	p-value	Positive, n (%)	OR	p-value	Positive, n (%)	OR	p-value
DEC^1^	89 (28.7)	62.01	<0.001	58 (38.7)	46.96	<0.001	13 (32.5)	9.14	0.005
STEC	30 (9.7)	16.49	<0.001	42 (28.0)	4.08	0.210	6 (15.0)	15.26	0.060
EPEC	48 (15.5)	28.19	<0.001	22 (14.7)	25.60	0.001	12(30.0)	35.52	0.010
EHEC	14 (4.5)	7.28	0.009	21 (14.0)	2.01	0.560	0 (0.0)	NA	NA
EAEC	14 (4.5)	7.28	0.009	4 (2.7)	4.08	0.210	0 (0.0)	NA	NA
O157	10 (3.2)	5.13	0.030	7 (4.7)	7.29	0.060	3 (7.5)	7.56	0.180
*Shigella*	43 (13.9)	24.78	<0.001	5 (3.3)	5.13	0.130	10 (25.0)	27.88	0.020
Rotavirus	54 (17.4)	32.46	<0.001	4 (2.7)	4.08	0.210	NA	NA	NA
Adenovirus	12 (3.9)	6.20	0.010	7 (4.7)	7.29	0.060	0 (0.0)	NA	NA
*Cryptosporidium*	12 (3.9)	6.20	0.010	15 (10.0)	16.55	0.006	2 (5.0)	5.25	0.280
*Giardia*^2^	2 (0.6)	1.00	-	1 (0.7)	1.00	-	3 (7.5)	7.56	0.180

OR, odds ratio; DEC, diarrhoeagenic *Escherichia coli*; STEC, Shiga toxin-producing *E. coli*; EPEC, enteropathogenic *E. coli*; EHEC, enterohemorrhagic *E. coli*; EAEC, enteroaggregative *E. coli*; NA, not applicable.

1 The chi-square statistic was calculated using a 2×2 contingency table; a similar analysis was carried out previously by Daniels et al. [32].

2 For humans and animals, the *Giardia* samples were used as reference.

**Table 2.** Distribution of different diarrhoeal pathogens in commonly domesticated animals

Infectious agents	Animal host		
	Sheep (n=60)	Cattle (n=45)	Goats (n=45)
DEC	25 (41.7)	15 (33.3)	16 (35.5)
STEC	19 (31.7)	12 (26.7)	12 (26.7)
EPEC	11 (18.3)	3 (6.7)	8 (17.8)
EHEC	11 (18.3)	4 (8.9)	6 (13.3)
EAEC	3 (5.0)	0 (0.0)	1 (2.2)
157	0 (0.0)	3 (6.7)	4 (8.9)
*Shigella*	4 ((6.7)	0 (0.0)	1 (2.2)
Rotavirus	3 (5.0)	1 (2.2)	0 (0.0)
Adenovirus	3 (5.0)	3 (6.7)	2 (4.4)
*Cryptosporidium*	2 (3.3)	5 (11.1)	8 (17.8)
*Giardia*	0 (0.0)	0 (0.0)	1 (2.2)

Values are presented as number (%). DEC, diarrhoeagenic *Escherichia coli*; STEC, Shiga toxin-producing *E. coli*; EPEC, enteropathogenic *E. coli*; EHEC, enterohemorrhagic *E. coli*; EAEC, enteroaggregative *E. coli*.

**Table 3.** Distribution by age of diarrhoeal pathogens detected in humans and animals

Infectious agent	Humans		Animals	
	≤2 yr (n=238)	>2 yr (n=78)	Young (n=35)	Adult (n=115)
DEC	49 (20.6)	40 (51.3)	12 (34.3)	46 (40.0)
STEC	15 (6.3)	15 (19.2)	8 (22.8)	34 (29.6)
EPEC	34 (14.3)	14 (17.9)	5 (14.3)	17 (14.8)
EHEC	10 (4.2)	4 (5.1)	5 (14.3)	16 (13.9)
EAEC	9 (3.8)	5 (6.4)	1 (2.8)	3 (2.6)
O157	8 (3.4)	2 (2.6)	0 (0.0)	6 (5.2)
*Shigella*	32 (13.4)	11 (14.1)	1 (2.8)	4 (3.5)
Rotavirus	47 (19.7)	7 (9.0)	2 (5.7)	2 (1.7)
Adenovirus	12 (5.0)	0 (0.0)	2 (5.7)	5 (4.3)
*Cryptosporidium*	6 (2.5)	6 (7.7)	4 (11.4)	11 (9.6)
Giardia	1 (0.4)	1 (1.3)	1 (2.8)	0 (0.0)

Values are presented as number (%). DEC, diarrhoeagenic *Escherichia coli*; STEC, Shiga toxin-producing *E. coli*; EPEC, enteropathogenic *E. coli*; EHEC, enterohemorrhagic *E. coli*; EAEC, enteroaggregative *E. coli*.

**Table 4.** Simultaneous detection of different diarrhoeal pathogens in faeces and soil samples

Infectious pattern	Human (n=152)	Animal (n=67)	Soil (n=16)
Single agent	92 (60.5)	26 (38.8)	3 (18.7)
Multiple agents	60 (39.5)	41(61.2)	13 (81.2)
p-value (odds ratio)	0.003 (1.75)	0.030 (1.79)	0.009 (5.90)
Major coinfections			
*Cryptosporidium*+STEC	2 (1.3)	7 (10.4)	0 (0.0)
EHEC+STEC	3 (2.0)	15 (22.4)	0 (0.0)
STEC+EPEC	9 (5.9)	13 (19.4)	5 (31.2)
Adenovirus+STEC	1 (0.6)	7 (10.4)	0 (0.0)
*Shigella*+STEC	13 (8.5)	3 (4.5)	3 (18.7)
*Shigella*+EPEC	10 (6.6)	3 (4.5)	6 (37.5)
Rotavirus+*Cryptosporidium*	1 (0.6)	3 (4.5)	0 (0.0)
Rotavirus+EPEC	11 (7.2)	0 (0)	0 (0.0)

Values are presented as number (%). STEC, Shiga toxin-producing *Escherichia coli*; EHEC, enterohemorrhagic *E. coli*; EPEC, enteropathogenic *E. coli*.
