# Supplementary material for: Major enteropathogens in humans, domestic animals, and environmental soil samples from the same locality: prevalence and transmission considerations in coastal Odisha, India
Source: Epidemiol Health. 2020 May 26;42:e2020034. doi: 10.4178/epih.e2020034 (PMC7644938; doi:10.4178/epih.e2020034)

Volume: 42, Article ID: e2020034
https://doi.org/10.4178/epih.e2020034

Supplementary Material 1: Detailed description of pathogen specific polymerase chain reaction primers for their specific detection

Pathogen	Target gene	Primer Name	Primer Sequence (5' to 3')	Tm (^0C)	Size (bp)
			Escherichia coli		
EPEC	eae	EPEC_eaeA_F	GACCCGGCACAAGCATAAGC	59	384
		EPEC_eaeA_R	CCACCTGCAGCAACAAGAGG		
EHEC	hlyA	EHEC_hlyA_F	GCATCATCAAGCGTACGTTCC	57	534
		EHEC_hlyA_R	AATGAGCCAAGCTGGTTAAGCT	54	180
EAEC	pCVD432 plasmid	EAEC_pCVD432_F	CTGGCGAAAGACTGTATCAT	51	630
		EAEC_pCVD432_R	AAATGTATAGAAATCCGCTGTT		
STEC	stx1	*E.coli*_stx1_F	ATAAATCGCCATTCGTTGACTAC		
		*E.coli*_stx1_R	GGCACTGTCTGAAACTGCTCC		
STEC	stx2	*E.coli*_stx2_F	GGCACTGTCTGAAACTGCTCC	56	255
		*E.coli*_stx2_R	TCGCCAGTTATCTGACATTCTG		
STEC	H7 FLIC	H7_FLIC-F	GCGCTGTCGAGTTCTATCGAGC	59	625
		H7_FLIC-R	CAACGGTGACTTATCGCCATTCC		
			***Shigella* Spp.**		
	ShET 1	*Shigella*_1A_F	CAG CGT CTT TCA GCG ACA GTG TTT	59	530
		*Shigella*_1A_R	AGC ATG ATA CTC AAC AGC CAG ACC		
	ShET 2	*Shigella*_1B_F	ATA CTG GCT CCT GTC ATT CAC GGT		
		*Shigella*_1B_R	GGA AGT GAC AGG GCA TTT GTG GAT		
			Cryptosporidium Spp.		
	18s SSU rRNA locus	18s Morgan F	AGTGACAAGAAATAACAATACAGG	60	298
		18s Morgan R	CCTGCTTTAAGCACTCTAATTTTC		
			Giardia Spp.		
	gdh gene	GDHeF	TCAAGCTYAAYGCYGGYTTGCGT	57	432
		GDHiF	CAGTACAACTCYGCTCTCGG		
		GDHiR	GTTRTCCTTGCACATCTCC		

Volume: 42, Article ID: e2020034
https://doi.org/10.4178/epih.e2020034

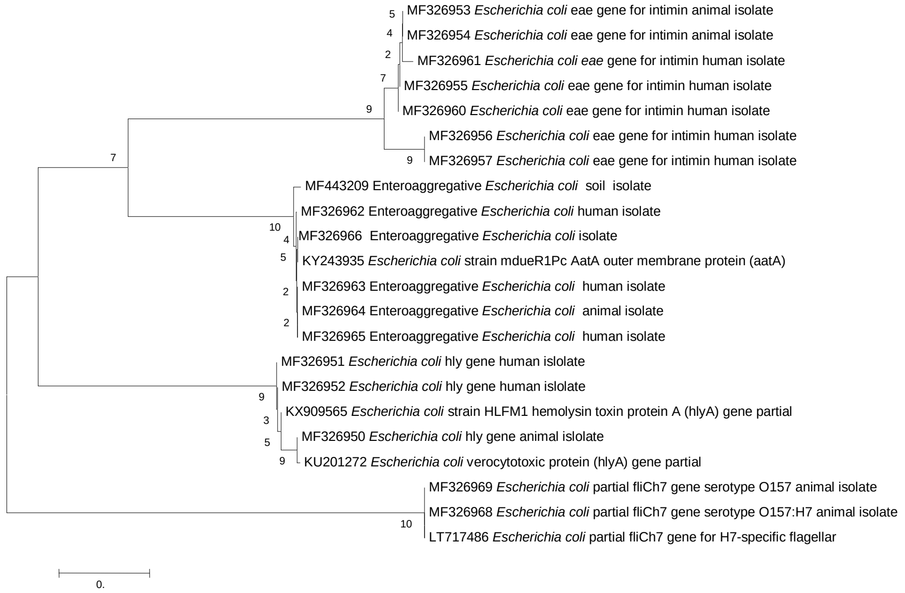

Supplement: Supplementary Material 1. [file epih-42-e2020034-suppl1.pdf]
