# Supplementary material for: Major enteropathogens in humans, domestic animals, and environmental soil samples from the same locality: prevalence and transmission considerations in coastal Odisha, India
Source: Epidemiol Health. 2020 May 26;42:e2020034. doi: 10.4178/epih.e2020034 (PMC7644938; doi:10.4178/epih.e2020034)

Volume: 42, Article ID: e2020034
https://doi.org/10.4178/epih.e2020034

Supplementary Material 2: Neighbor-joining tree for Genetic relatedness of pathogenic *E. coli* strains identified in this study; The scale indicates the number of nucleotide substitutions per position. Numbers above the branches indicate bootstrap percentages (those above 50%) based on 1,000 replicates. Reference strains of pathogenic *E. coli* were selected from GenBank, using the accession numbers indicated in the text

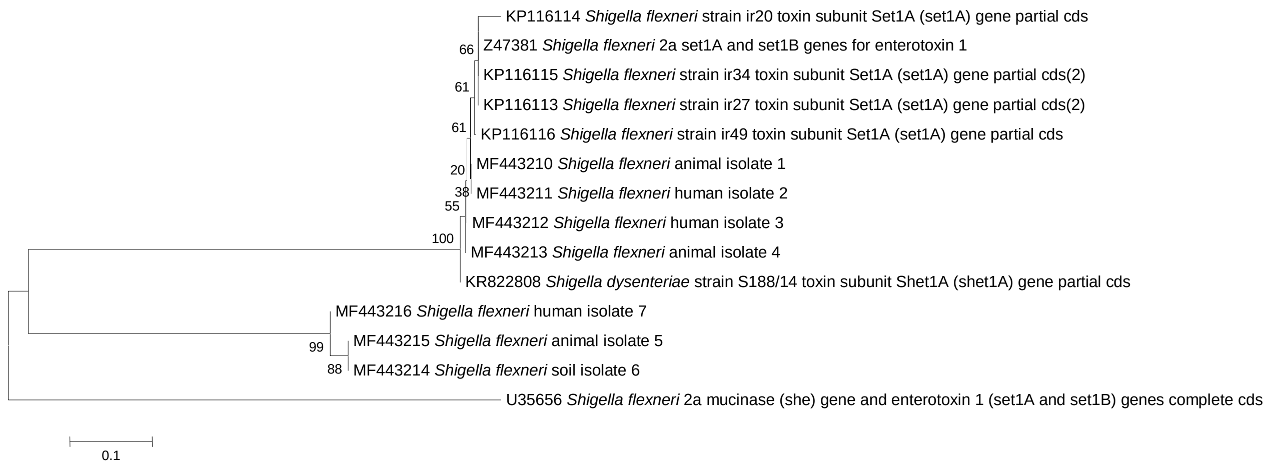

Supplement: Supplementary Material 2. [file epih-42-e2020034-suppl2.pdf]
